# Chronopharmacokinetics of once daily dosed aminoglycosides in hospitalized infectious patients

**DOI:** 10.1007/s11096-015-0066-7

**Published:** 2015-01-24

**Authors:** Erik van Maarseveen, Wai Hong Man, Johannes Proost, Cees Neef, Daniël Touw

**Affiliations:** 1 Department of Clinical Pharmacy, University Medical Center Utrecht, Heidelberglaan 100, 3584 CX Utrecht, The Netherlands; 2 Department of Pharmacokinetics, Faculty of Mathematic and Natural Sciences, University of Groningen, Broerstraat 5, 9700 AB Groningen, The Netherlands; 3 Department of Clinical Pharmacy and CAHPRI, Maastricht University Medical Center, P. Debyelaan 25, 6229 HX Maastricht, The Netherlands; 4 Department of Clinical Pharmacy and Pharmacology, University Medical Center Groningen, P.O. Box 30.001 (internal post code EB70), 9700 RB Groningen, The Netherlands; 5 Department of Clinical Pharmacy, University Medical Center Utrecht, Room HP D00.218, Heidelberglaan 100, P.O. Box 85500, 3508 GA Utrecht, The Netherlands

**Keywords:** Aminoglycosides, Circadian, Diurnal, Gentamicin, Nephrotoxicity, Pharmacokinetics, Tobramycin

## Abstract

*Background* hospitalized patients with serious infections treated with aminoglycosides are at risk of developing nephrotoxicity. Previous clinical studies have shown that the pharmacokinetics of aminoglycosides in humans follow a circadian rhythm. Therefore, the time of administration could have important clinical implications with respect to the risk of developing aminoglycoside-associated nephrotoxicity in patients treated with once daily dosing regimens. *Objective* To examine the effect of the time period of administration on aminoglycoside exposure and the incidence of nephrotoxicity in a large population of hospitalized patients with serious infections. *Setting* General ward and intensive care unit of a teaching hospital. *Method* In this retrospective cohort study, patients treated with intravenous tobramycin or gentamicin were eligible for inclusion. Patients were divided into three groups by time of administration: morning, afternoon and night. *Main outcome measure* Pharmacokinetic parameters and the incidences of nephrotoxicity were compared between the morning, afternoon and evening groups. *Results* 310 general ward and 411 intensive care unit patients were included. No significant differences were found in patient characteristics between the morning, afternoon and night groups. The time period of administration did not affect aminoglycoside pharmacokinetics or the incidence of nephrotoxicity. *Conclusion* The time of administration has no effect on the pharmacokinetics or nephrotoxicity of once daily dosed aminoglycosides in hospitalized patients. Consequently, we advise aminoglycosides to be administered as soon as possible in case of (suspected) severe hospital-acquired infections and subsequent dosages to be based on therapeutic drug monitoring to optimize the efficacy/toxicity balance.

## Impacts on practice

- There is no effect of the diurnal rhythm on aminoglycoside pharmacokinetics hospitalized clinically infected patients.
- Administration of aminoglycosides during the nighttime (21:00–5:00) compared to the daytime (5:00–21:00) does not increase the risk of nephrotoxicity in hospitalized patients with serious infections.
- Shifting administrations of aminoglycosides from nighttime to daytime most likely does not offer any benefit to hospitalized patients with serious infections.

## Introduction

Aminoglycoside antibiotics remain an important group of antimicrobial agents in the treatment of serious infections by gram-negative organisms [1]. Although they are highly effective at killing these organisms, exposure to intravenous aminoglycosides comes with an increased risk of auditory and renal toxicity [2]. All hospitalized patients, especially critically ill patients with a low renal clearance of aminoglycosides, are at high risk of developing nephrotoxicity [3, 4]. In an attempt to reduce the nephrotoxic side effects without compromising efficacy, once-daily dosing regimens of aminoglycosides have shown to be superior to conventional multiple daily dosing regimens [5]. Nowadays, intravenous aminoglycosides are primarily dosed once daily [6]. Since a circadian rhythm of aminoglycoside pharmacokinetics has been observed in animal experiments [7–10] as well as in human studies involving multiple daily dosing protocols [11, 12], the clearance of aminoglycosides is expected to be higher during daytime compared to nighttime due to a higher glomerular filtration rate in the ‘active period’. As a result, the time of administration in patients receiving their drug with 24-h intervals could have important implications with respect to aminoglycoside clearance. Nevertheless, human data on the effect of the circadian rhythm on the pharmacokinetics and toxicity of once daily dosed aminoglycosides are inconsistent [13, 14]. To date, the effect of diurnal changes on aminoglycoside pharmacokinetics has not been extensively studied in a large cohort of patients with serious infections.

## Aim of the study

To examine the effect of time period of administration on aminoglycoside pharmacokinetics and the incidence of nephrotoxicity in a large population of hospitalized clinically infected patients.

## Ethical approval

A waiver for consent was provided by the local medical ethics committee.

## Method

In this retrospective cohort study data were extracted from patients’ clinical records and a therapeutic drug monitoring database. All general ward and intensive care unit patients of 18 years or older who were admitted to hospital between January 2006 and December 2013 and were treated with intravenous tobramycin or gentamicin were eligible for inclusion. Furthermore, an estimated glomerular filtration rate calculated with the MDRD formula [15] higher than 25 mL/min and a once daily dose of 4–6 mg/kg were required. Finally, two aminoglycoside serum concentrations drawn after the first or second infusion were mandatory for inclusion. For the analysis of nephrotoxicity in the general ward population, the treatment duration had to be 2 days or longer. Furthermore, a minimum of two serum creatinine concentrations was required; one drawn between 48 h before and 12 h after initiation of therapy. If two or more concentrations were available in this time frame the concentration drawn closest to the time of intiation of aminoglycoside therapy was selected as the baseline creatinine concentration. A second ‘post-treatment’ concentration drawn between 48 h after initiation and 48 h after cessation of therapy was mandatory for inclusion. If two ‘post-treatment’ concentrations were available in this period the highest creatinine concentration was appointed as the ‘post-treatment’ concentration. The delta serum creatinine (ΔScr) was defined by the ‘post-treatment’ change in creatinine serum concentration from baseline. Nephrotoxicity was defined as a minimal ΔScr of 45 nmol/mL (0.5 mg/dL) [14]. According to the therapeutic drug monitoring (TDM) protocol, a peak concentration and second concentration were drawn 0.5–1 and 6–14 h after end of the first or second infusion, respectively. The target peak concentration was 15–20 mg/L, and the target trough concentration was <0.5 mg/L. It was a priori decided to analyze the pharmacokinetics separately for general ward and intensive care unit populations and to analyze the incidence of nephrotoxicity only in the general ward population, because the kidney function of the latter population has an inherently large variability and many artifacts occur due to cardiovascular management. Patients were divided into three groups by time of administration: 5:00–12:55 (morning), 13:00–20:55 (afternoon) and 21:00–4:55 (night). Pharmacokinetic parameters were calculated for each individual patient using pharmacokinetic software (MW\Pharm 3.80, MediWare, Zuidhoorn, The Netherlands) and a population pharmacokinetic model.

### Statistical analyses

A Kolmogorov–Smirnov test was used to test normal distribution of the data. Means and standard deviations of continuous variables are presented in case of normal distribution. Otherwise, medians and ranges were reported. One-way ANOVA with Bonferroni post hoc tests were used to determine statistical differences among normal data distributions. If the data were not normally distributed, a Kruskal–Wallis non-parametric method was applied with Mann–Whitney-U post hoc tests including correction for multiple testing. A Chi square test was used to compare dichotomous variables between groups. All statistical analyses were performed using GraphPad Prism version 5.03 for Windows (GraphPad Software, San Diego, CA, USA). A *p* value of <0.05 was considered to be statistically significant and in each instance a two-sided test was carried out.

## Results

A total of 1,452 general ward and 843 intensive care unit patients were selected from the TDM database. Approximately half of the selected general ward and intensive care unit patients were excluded as a result of missing pharmacokinetic data. In the general ward population, subsequently again half of the remaining patients were excluded from the nephrotoxicity analysis because of missing creatinine values or a treatment duration shorter than 2 days. In total 310 general ward and 411 intensive care unit patients were eligible for inclusion in the morning, afternoon and night groups. No significant differences were found in patient characteristics between the three groups in both populations (Table 1).

**Table 1:** Patient characteristics

General ward	Time of administration			
	Morning (n = 88)	Afternoon (n = 108)	Night (n = 114)	*p* value
Age (years)	63 ± 34	62 ± 27	57 ± 31	0.56^a^
Weight (kg)	70 ± 29	71 ± 27	70 ± 26	0.33^a^
% of males	42	48	56	0.10^c^
% of patients on TOB	21	27	19	0.23^c^
eGFR (ml/min)	80 (26–191)	88 (26–189)	89 (26–185)	0.27^b^
Nephrotoxic comedication				0.26^c^
*Furosemide (%)*	*2.5*	*3.1*	*2.9*	
*Vancomycin (%)*	*1.1*	*1.2*	*3.1*	
Ward				0.42^c^
*Surgical (%)*	*39*	*40*	*34*	
*Internal (%)*	*53*	*51*	*49*	
*Pulmonary diseases (%)*	*6*	*7*	*12*	
*Other (%)*	*3*	*2*	*4*	

Intensive care unit	Morning (n = 124)	Afternoon (n = 145)	Night (n = 142)	*p* value
Age (years)	68 ± 32	65 ± 28	68 ± 31	0.14^a^
Weight (kg)	72 ± 28	75 ± 26	75 ± 24	0.48^a^
% of males	64	65	69	0.47^c^
% of patients on TOB	48	43	39	0.35^c^
eGFR (ml/min)	55 (25–167)	64 (25–183)	57 (25–167)	0.41^b^
SAPS II score, mean ± SD	40.7 ± 13.4	41.0 ± 13.2	44.6 ± 14.1	0.32^b^

Dichotomous variables are displayed as percentages. Continuous variables are presented as means ± SD or medians with ranges in parentheses (min–max). Abbreviations are: *eGFR* estimated glomerular filtration rate, *SAPS* simplified acute physiology score, *TOB* tobramycin

The italics indicate that furosemide, vancomycin etc are subgroups of nephrotoxic comedication. Likewise, surgical and internal are subgroups of Ward

^a^ANOVA

^b^Kruskall–Wallis test

^c^Chi square test

Although a trend toward a higher volume of distribution in the afternoon group was seen in the general ward population compared to the morning or night groups (0.28, 0.25 and 0.24 L/kg respectively, *p* = 0.06), aminoglycoside clearance and area-under-the-curve (AUC) did not differ significantly between the morning, afternoon and night groups in both populations (Table 2).

**Table 2:** Dosing, pharmacokinetics and kidney function parameters

General ward	Time of administration			
	Morning (n = 88)	Afternoon (n = 108)	Night (n = 114)	*p* value
Dose (mg)	350 (180–500)	360 (220–600)	350 (240–650)	0.48^b^
Treatment duration (days)	5 (3–18)	5 (3–22)	5 (3–21)	0.98^b^
CL (L/h)	4.4 ± 1.7	4.6 ± 1.8	4.7 ± 1.7	0.41^a^
Vd (L/kg)	0.25 ± 0.08	0.28 ± 0.09	0.24 ± 0.08	0.06^a^
AUC (mg h/L)	135 ± 47	139 ± 48	145 ± 51	0.97^a^
ΔScr (nmol/mL)	6 (−40–167)	0 (−11–323)	−1 (−56–467)	0.11^b^
Nephrotoxicity (%)	18	12	10	0.47^c^

ICU	Morning (n = 124)	Afternoon (n = 145)	Night (n = 142)	*p* value
Dose (mg)	350 (250–675)	360 (240–600)	360 (150–750)	0.36^b^
Treatment duration (days)	4 (3–12)	4 (3–13)	5 (3–17)	0.72^b^
CL (L/h)	1.9 ± 0.06	2.1 ± 0.08	2.1 ± 0.07	0.19^a^
Vd (L/kg)	0.33 ± 0.11	0.32 ± 0.10	0.33 ± 0.12	0.85^a^
AUC (mg h/L)	195 ± 71	183 ± 75	173 ± 67	0.86^a^

Dichotomous variables are displayed as percentages. Continuous variables are presented as means ± SD or medians with ranges in parentheses (min–max). Abbreviations are: *AUC* area under the concentration time curve, *CL* clearance, *Scr* serum creatinine, *Vd* volume of distribution, *ΔScr* median delta serum creatinine concentration

^a^ANOVA

^b^Kruskall–Wallis test

^c^Chi square test

Finally, no significant differences in ΔScr (*p* = 0.11) or in percentages of patients developing nephrotoxicity (*p* = 0.47) were found between the morning, afternoon and night groups of the general ward population (Table 2).

## Discussion

This large retrospective study found no differences in aminoglycoside exposure or nephrotoxicity related to time of administration in hospitalized patients with serious infections. Caution needs to be exercised when interpreting the results of any retrospective cohort study because of errors due to confounding and inclusion bias. However, baseline characteristics showed no significant differences between groups and the proportion of excluded patients per exclusion criterion did not differ significantly between patients who were administered aminoglycosides in the morning, afternoon and night.

Since most previous studies did find an effect of the circadian rhythm on the pharmacokinetics of aminoglycosides, our results intuitively seem unexpected. Nevertheless, an in-depth review of the available evidence demonstrates that the animal and preclinical studies were performed in relatively small populations of animals or (young) healthy volunteers, primarily investigated multiple daily or even continuously dosed aminoglycoside dosing regimens, had suboptimal pharmacokinetic sampling protocols with only peak and trough concentration monitoring and/or reported on amikacin and kanamycin that are more likely to accumulate [7–12, 16–18]. In addition and perhaps most eminent, TDM in the majority of these studies was performed after 3 days of therapy. At that moment in time a large proportion of patients will no longer show signs of an infection and aminoglycosides are withdrawn in current clinical practice. It is thus critical to collect pharmacokinetic data during the first 2 days of therapy when the patient is still showing signs of an ongoing infection, since sepsis and an altered status of the immune system disrupt the circadian rhythm [19, 20]. Our study investigated a large population of hospitalized patients with serious infections, who received their aminoglycosides once daily with optimal pharmacokinetic sampling during the first or the second administration.

The results from human studies examining the effect of the circadian rhythm on the pharmacokinetics of aminoglycosides are conflicting [14, 21–24]. The three ‘positive’ studies all involved multiple daily dosing protocols of ‘earlier’, and more accumulating aminoglycosides (e.g. kanamycin and amikacin). In contrast, the two ‘negative’ studies reported on once or twice daily dosed aminoglycosides that show relatively low tissue accumulation (e.g. tobramycin, netelmicin and gentamicin). A randomized controlled trial by Fauvelle et al. [23], in which netilmicin (4.5 mg/kg of lean body weight) was administered every 24 h at 10 a.m. or 10 p.m. to 23 ill febrile patients with severe infection, showed that the time of administration had no influence on netilmicin concentrations in serum nor on pharmacokinetic parameters in these patients. The second was a prospective cohort study by Prins et al. [14] who investigated the influence of the time of drug administration on serum drug concentrations and the incidence of nephrotoxicity in 179 patients with serious infections treated with gentamicin or tobramycin once daily. This study found no statistically significant differences in trough or peak concentrations for three time periods of administration. Nevertheless, the incidence of nephrotoxicity was significantly higher when the aminoglycosides were administered during the nighttime (midnight to 7:30 AM; *p* = 0.004) to a combined general ward and intensive care unit population. In our opinion, data on pharmacokinetics and nephrotoxicity of aminoglycosides in general ward and intensive care unit populations should be analyzed separately since admission to the intensive care unit is a known risk factor for acute kidney injury [25, 26]. Moreover, the study by Prins et al. [14] demonstrated that intensive care unit patients are more ‘at risk’ to receive their aminoglycosides during the night (77 vs. 44 %). Admission to the intensive care unit is thereby a confounder by definition, since it correlates with both the dependent (i.e. nephrotoxicity) and the independent variable (i.e. time of administration). Furthermore, the authors had to statistically correct for not only the higher intensive care unit admissions but also for significantly poorer baseline creatinine clearance (84 vs. 100 mL/min, *p* < 0.05) in the nighttime compared to daytime group. All of the above are established risk factors of acute kidney injury [25, 26]. Altogether, it is debatable whether statistical correction is a proper tool to adjust for these imbalances in patient characteristics.

Our data show a trend of a higher volume of distribution in the afternoon group of the general ward population but not in the intensive care unit population. This is most likely caused by the discrepancy in the daily fluid management of a general ward patient compared to an intensive care unit patient. Whereas the latter will receive intravenous fluids continuously, contributing to a relatively stable volume of distribution throughout the day, a general ward patient will gain fluids during the day causing the volume of distribution of hydrophilic aminoglycosides to peak in the afternoon.

The presented pharmacokinetic data are in accordance with the data reported by Fauvelle et al. [23] and are further supported by the unpublished results of two randomized clinical trials in adult and pediatric cystic fibrosis patients suffering from pulmonary exacerbation. Both studies found no effect of the circadian rhythm on the pharmacokinetics of once daily dosed tobramycin in cystic fibrosis patients (submitted for publication).

## Conclusion

Currently, it is widely believed that administration of aminoglycosides in the morning will result in a reduction in nephrotoxicity and many (international) antibiotic guidelines advise administration of aminoglycosides in the morning. This large retrospective study, however, shows no administration-time-related differences in aminoglycoside exposure or nephrotoxicity in hospitalized clinically infected patients. It is therefore unlikely that administration during the nighttime will result in a higher incidence of renal toxicity in these patients. We recommend aminoglycosides to be administered as soon as possible in case of (suspected) severe hospital-acquired pneumonia or sepsis to reduce the pen-to-needle time in antibiotics, which has shown to be effective in preventing death [27]. Subsequent doses should be based on therapeutic drug monitoring to optimize the efficacy/toxicity balance.
